# Supplementary material for: Bactericidal Effect of Clove Oil against Multidrug-Resistant Streptococcus suis Isolated from Human Patients and Slaughtered Pigs
Source: Pathogens. 2019 Dec 21;9(1):14. doi: 10.3390/pathogens9010014 (PMC7169397; doi:10.3390/pathogens9010014)
Supplement: Supplementary file 1 [file pathogens-09-00014-s001.zip › S1-Supplement data for time kill assay.pdf]

S. suis MNCM06	Time (min)	Bacterial count (log cfu/mL)				
		#1	#2	#3	Average	SD
Control	0	6.650	6.660	6.600	6.637	0.032
	5	6.600	6.620	6.640	6.620	0.020
	10	6.580	6.600	6.610	6.597	0.015
	15	6.540	6.480	6.560	6.527	0.042
	30	6.460	6.470	6.460	6.463	0.006
	60	6.320	6.410	6.380	6.370	0.046
Treated	0	5.900	5.900	5.900	5.900	0.000
	5	3.450	3.460	3.410	3.440	0.026
	10	2.040	2.000	2.040	2.027	0.023
	15	0.000	0.000	0.000	0.000	0.000
	30	0.000	0.000	0.000	0.000	0.000
	60	0.000	0.000	0.000	0.000	0.000
