# Supplementary material for: Bactericidal Effect of Clove Oil against Multidrug-Resistant Streptococcus suis Isolated from Human Patients and Slaughtered Pigs
Source: Pathogens. 2019 Dec 21;9(1):14. doi: 10.3390/pathogens9010014 (PMC7169397; doi:10.3390/pathogens9010014)
Supplement: Supplementary file 1 [file pathogens-09-00014-s001.zip › S2-Supplement data for effect of pH on bactericidal activity of clove oil.pdf]

S. suis MNCM06	Time (min)	Bacterial count (log cfu/mL)					
		#1	#2	#3	average	STDEV	Standard Error
pH4	0	5.176	5.180	5.180	5.179	0.002	0.001
	5	3.180	3.180	3.146	3.169	0.020	0.011
	10	2.000	2.000	2.000	2.000	0.000	0.000
	15	0.000	0.000	0.000	0.000	0.000	0.000
	30	0.000	0.000	0.000	0.000	0.000	0.000
	60	0.000	0.000	0.000	0.000	0.000	0.000
pH5.5	0	5.114	5.146	5.130	5.130	0.016	0.009
	5	3.826	3.810	3.810	3.815	0.009	0.005
	10	2.180	2.200	2.180	2.187	0.012	0.007
	15	0.000	0.000	0.000	0.000	0.000	0.000
	30	0.000	0.000	0.000	0.000	0.000	0.000
	60	0.000	0.000	0.000	0.000	0.000	0.000
pH7	0	5.398	5.410	5.398	5.402	0.007	0.004
	5	3.672	3.672	3.680	3.675	0.005	0.003
	10	2.380	2.380	2.380	2.380	0.000	0.000
	15	0.000	0.000	0.000	0.000	0.000	0.000
	30	0.000	0.000	0.000	0.000	0.000	0.000
	60	0.000	0.000	0.000	0.000	0.000	0.000
pH8	0	5.447	5.407	5.412	5.422	0.022	0.013
	5	3.750	3.756	3.750	3.752	0.003	0.002
	10	2.300	2.300	2.316	2.305	0.009	0.005
	15	0.000	0.000	0.000	0.000	0.000	0.000
	30	0.000	0.000	0.000	0.000	0.000	0.000
	60	0.000	0.000	0.000	0.000	0.000	0.000
